# Reassessing re‐TURBT in the modern era: Is it time to move from routine to selective?

**DOI:** 10.1002/bco2.70269

**Published:** 2026-08-20

**Authors:** Mithilesh Yadav, Swarnendu Mandal, Sambit Tripathy, Pranjal Chowdhury, Vivek Rathod, Manoj Kumar Das, Kalandi Barik, Prasant Nayak

**Affiliations:** ^1^ Department of Urology and Renal Transplant All India Institute of Medical Sciences (AIIMS) Bhubaneswar India

**Keywords:** non‐muscle‐invasive bladder cancer, repeat transurethral resection (re‐TURBT), residual tumour, risk‐adapted approach, selective Re‐TURBT, upstaging

## Abstract

**Objectives:**

This work aimed to determine the pathological yield of restaging transurethral resection of bladder tumour (re‐TURBT) in patients with non‐muscle‐invasive bladder cancer (NMIBC) managed at a contemporary tertiary‐care setting and to describe clinicopathological features among patients with positive restaging histopathology to select patients most likely to benefit from re‐TURBT.

**Patients and Methods:**

This single‐centre retrospective observational case–control analysis within a re‐TURBT cohort included patients who underwent macroscopically complete index TURBT for presumed NMIBC and subsequently underwent restaging TURBT within the institutional pathway. Patients with muscle‐invasive disease at index TURBT, benign pathology, non‐urothelial carcinoma, variant histology, or ineligibility for re‐TURBT for any reason were excluded. The primary outcome was positive restaging histopathology, defined as residual urothelial neoplasia or pathological progression. In addition, clinicopathological variables were explored to identify factors potentially associated with positive re‐TURBT, including tumour size, multifocality, age, sex, tumour grade, detrusor muscle status, and carcinoma in situ on index histopathology.

**Results:**

Among 188 TURBT records assessed, 79 patients underwent re‐TURBT and formed the analysed cohort. Median age was 64 years (IQR 56–69), 69 patients (87.3%) were male, and detrusor muscle was present in 77 index specimens (97.5%). Positive restaging histopathology occurred in nine patients (11.4%), comprising residual disease in eight (10.1%) and Ta high‐grade to T1 high‐grade progression in one (1.3%); no muscle‐invasive upstaging was observed. In the T1‐only analysis, positivity occurred in 8/69 (11.6%). Larger tumours, multifocality and CIS were more frequent among positive cases. Follow‐up recurrence admission occurred in 9 patients (11.4%).

**Conclusion:**

In this contemporary re‐TURBT series, the pathological yield of restaging was low, with uncommon progression. Positivity clustered in those with larger tumours (≥3 cm) and high‐risk biology (multifocal disease and carcinoma in situ on index histopathology). These findings are hypothesis‐generating and support prospective evaluation of selective restaging strategies in the contemporary era.

## 1 INTRODUCTION

Transurethral resection of bladder tumour remains the cornerstone of diagnosis, staging and initial management for non‐muscle‐invasive bladder cancer. A good‐quality index TURBT should provide representative tissue for histological grade and stage, document the presence or absence of detrusor muscle, and achieve complete macroscopic clearance whenever feasible. Despite its central role, index resection can be limited by several procedural and pathological factors, potentially leading to residual tumour and inaccurate staging. Fragmentation of tissue during piecemeal resection, cautery artefact, suboptimal visualisation, bleeding, and difficulty in completely excising a broad tumour base can all contribute to disease persistence. On the pathological side, the depth of the submitted specimen and the clarity of invasion reporting directly influence staging accuracy. Residual tumour at repeat resection is clinically relevant because it can increase the risk of early recurrence, compromise subsequent intravesical treatment planning, and obscure the presence of more advanced disease than initially suspected.

For these reasons, restaging transurethral resection of bladder tumour became embedded in practice, especially for stage T1 and high‐grade disease, for incomplete resections, and when the detrusor muscle was absent in the index specimen. Contemporary guidance continues to recommend early repeat resection for stage T1 disease, largely based on older series demonstrating high rates of residual tumour and clinically meaningful upstaging that altered management.^1^, ^2^, ^3^ However, contemporary practice has undergone substantial evolution. The increasing emphasis on resection quality, improved imaging adjuncts, and systematic attention to sampling depth have raised the possibility that the incremental benefit of routine restaging may be diminishing in certain contexts. Contemporary systematic reviews suggest that, while residual tumour after repeat resection remains common in selected high‐risk cohorts, the absolute yield varies widely and may be lower in centres with high‐quality index resections.^4^, ^5^, ^6^ Repeat resection is not a benign intervention; it entails an additional anaesthetic and operative episode, increases perioperative morbidity, may delay initiation of intravesical therapy, and adds substantial healthcare costs, underscoring the importance of defining subgroups most likely to benefit.

We conducted a single‐centre retrospective observational case–control analysis within a re‐TURBT cohort to assess the contemporary pathological yield of restaging transurethral resection after index TURBT and to describe the clinicopathological characteristics of patients with positive restaging histopathology. The analysis compared patients with positive and negative restaging findings, focusing on clinically relevant variables including tumour multiplicity, tumour size, age, sex, and the presence of carcinoma in situ.

## 2 PATIENTS AND METHODS

This is a retrospective observational case–control analysis within a re‐TURBT cohort that includes all patients who underwent an index transurethral resection of a bladder tumour at a tertiary centre between January 2021 and October 2025 (*n* = 188). From this pool, 79 patients formed the final study cohort, who subsequently underwent restaging transurethral resection of bladder tumour within the institutional pathway, in accordance with contemporary European Association of Urology and American Urological Association recommendations for repeat resection in eligible non‐muscle‐invasive bladder cancer.

### 2.1 Patient selection

Institutional TURBT records were screened to identify patients who underwent a macroscopically complete index TURBT for presumed NMIBC and subsequently underwent a re‐TURBT, in accordance with institutional practice and contemporary EAU and AUA guideline‐based eligibility criteria. The inclusion criteria were: index TURBT for suspected urothelial NMIBC, documented complete macroscopic clearance at index resection, eligibility for restaging TURBT according to guideline or institutional criteria, and completion of re‐TURBT with available histopathology.

**FIGURE 1:**
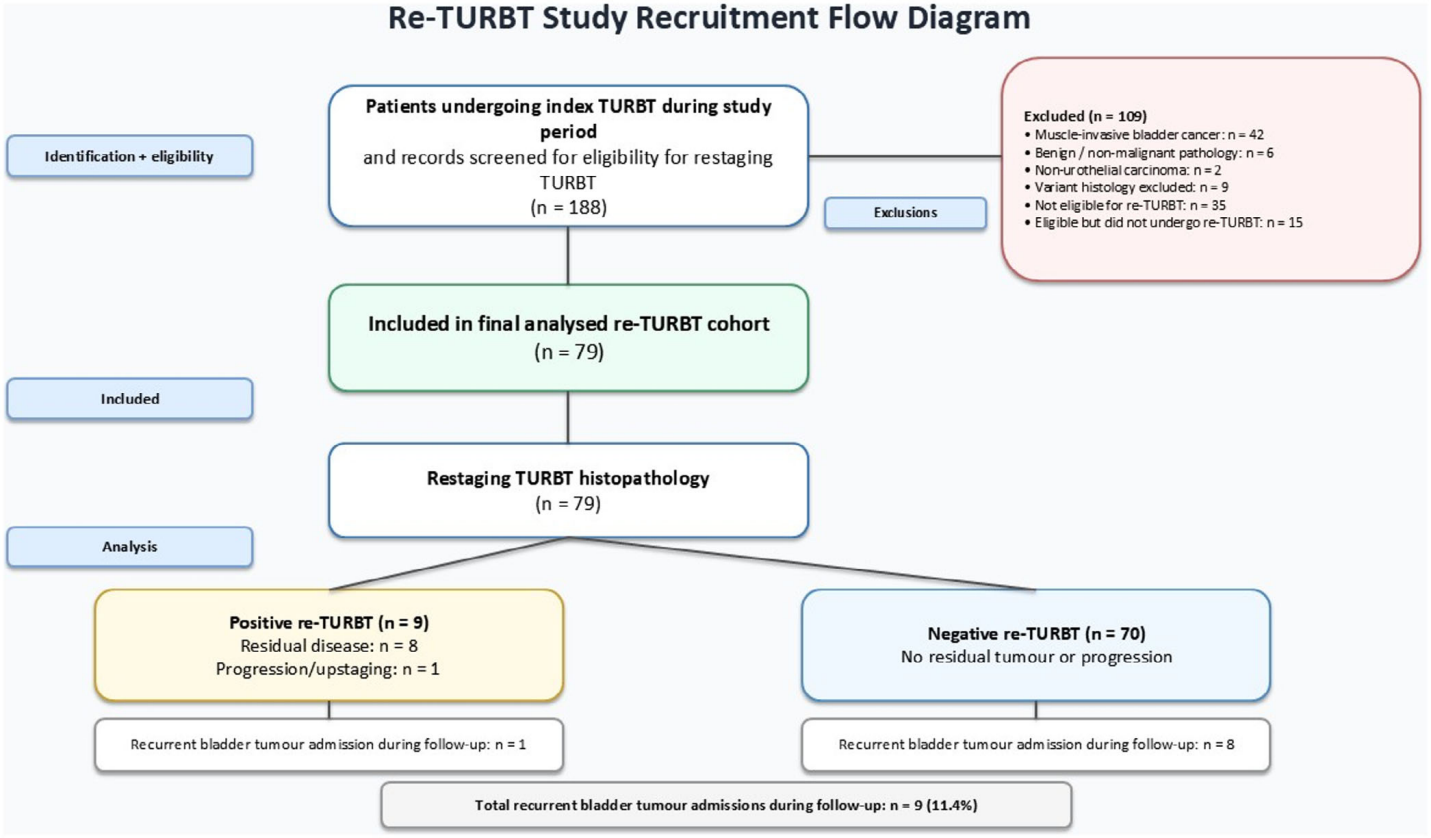
Study recruitment flow diagram.

Exclusion criteria were muscle‐invasive bladder cancer at index TURBT, benign or nonmalignant pathology, nonurothelial carcinoma, excluded variant histology, absence of guideline/institutional eligibility for re‐TURBT, and eligible disease in which re‐TURBT was not performed. As shown in Figure 1, of the 188 TURBT records assessed, 42 patients were excluded for muscle‐invasive disease at index TURBT, six for benign/nonmalignant pathology, two for nonurothelial carcinoma, nine for excluded variant histology, 35 because they were not eligible for re‐TURBT as per guideline/institutional criteria, and 15 because they were eligible but did not undergo re‐TURBT. Among the 15 eligible patients who did not undergo re‐TURBT, nine refused or were lost to follow‐up and six underwent early radical cystectomy. The final analysed cohort included 79 patients.

### 2.2 Index TURBT and restaging TURBT technique

Index TURBT was performed within a tertiary urology unit using systematic cystoscopy, tumour mapping, and complete macroscopic resection whenever technically feasible. White‐light cystoscopy with narrow‐band imaging was performed on the Olympus OTV‐S300 as part of institutional practice. Tumour number, size and site were recorded. A separately labelled deep resection or deep muscle biopsy was taken to include detrusor muscle, and targeted biopsies were obtained from suspicious areas when carcinoma in situ (CIS) was suspected. HolERBT and bipolar energy were used as per standard practice for index TURBT, and intraoperative details were recorded.

Restaging TURBT was generally performed within 2 to 6 weeks of the index TURBT. The restaging procedure included mandatory resection of the previous resection scar and tumour base with detrusor muscle sampling, along with resection or biopsy of any visible residual lesion. Specimens were submitted in separately labelled containers when applicable. All index and restaging transurethral resections were performed either by consultant urologists or by urology residents under direct consultant supervision. Each supervising consultant had approximately 10 years of endourology experience, ensuring procedural oversight and consistency in operative practice. However, surgeon‐specific caseload, individual operator experience, and surgeon‐level outcomes were not separately captured in the retrospective database and therefore could not be analysed. Intravesical therapy was administered according to risk category and institutional protocol, but treatment‐completion data were not analysed as a treatment exposure in this descriptive study.

### 2.3 Definitions and outcomes

The primary pathological outcome was positive restaging histopathology, defined as residual urothelial neoplasia or pathological progression compared with the index TURBT. Residual disease refers to persistent urothelial neoplasia at re‐TURBT without an increase in pathological stage. Progression was defined as a higher pathological stage or grade on re‐TURBT compared with the index specimen. The prespecified descriptive variables were age, sex, index stage, tumour grade, tumour size (<3 cm vs. ≥3 cm), tumour multiplicity, detrusor muscle presence in the index specimen, and CIS on index histopathology.

Restaging transurethral resection was performed within 2 to 6 weeks of the index procedure and included mandatory resection of the previous resection scar and base with detrusor muscle, along with resection or biopsy of any visible residual lesion, with specimens submitted in separate labelled containers (tumour, base, and additional biopsies when clinically indicated). Postoperative intravesical therapy was administered according to risk category and institutional protocol, including immediate mitomycin C where appropriate and bacillus Calmette–Guérin or intravesical chemotherapy schedules when indicated.

For pathological staging in this study, classification was based on the final impression of the histopathology report. Presence of detrusor muscle (deep muscle/muscularis propria) in the index transurethral resection specimen was specifically recorded and used as a surrogate marker of adequacy of the index resection; deep muscle was present in almost all specimens. Histopathological parameters documented for both the index and restaging resections included depth of invasion, tumour grade, variant histology (with percentage where reported), lymphovascular invasion, and the presence of carcinoma in situ.

The protocol used an explicit hypothesis‐testing design against historical benchmarks describing high residual and upstaging rates after initial resection.^4^, ^5^, ^7^ Sample size was calculated for the composite primary pathological yield endpoint of positive re‐TURBT, defined as residual urothelial tumour and/or pathological progression/upstaging at restaging TURBT. As residual tumour represents the dominant and more frequently observed component of this composite endpoint, the calculation was based on residual tumour yield. Historical literature reports residual tumour on repeat TURBT in approximately 20%–71% of patients with T1 disease, with older series reporting rates around 50%, including 53% in T1 patients in the study by Grimm et al.^4^, ^5^ In contrast, Yanagisawa et al. reported a contemporary pooled residual tumour rate of 31.4% among T1 patients undergoing conventional TURBT in the 2010s.^8^ Using a one‐sample proportion framework to compare a historical benchmark rate of 50% with a contemporary expected rate of 31.4%, with a two‐sided alpha of 0.05 and 90% power, 72 evaluable patients were required. After allowing for approximately 10% nonevaluable records or loss to follow‐up, the target sample size was 80 patients. The final analysed cohort included 79 patients, satisfying the required evaluable sample size and closely approximating the planned target sample size. This calculation was intended for the pathological yield endpoint and was not intended to power exploratory clinicopathological comparisons or predictor modelling.

Analyses were conducted using contingency tables with Fisher's exact test for categorical variables. Effect sizes were summarised using odds ratios with 95% confidence intervals. Age was examined as a continuous variable per 10‐year increment and was additionally dichotomised at >70 years, as this threshold is recognised in contemporary NMIBC risk‐stratification frameworks as an additional clinical risk factor.

## 3 RESULTS

Seventy‐nine patients underwent transurethral resection of bladder tumour, followed by restaging transurethral resection, and were included in the analysis. Baseline demographics and tumour characteristics are summarised in Table 1. The cohort had a mean age of 62.6 ± 10.6 years and a median age of 64 years (interquartile range, 56–69 years). There were 69 males (87.3%) and 10 females (12.7%). On index histopathology, stage was classified as Ta in 10 patients (12.7%) and T1 in 69 patients (87.3%). The detrusor muscle was present in 77 index specimens (97.5%) and not identified in two (2.5%). Tumour size was <3 cm in 46 patients (58.2%) and ≥3 cm in 33 (41.8%). A single tumour was present in 55 patients (69.6%), whereas multiple tumours were documented in 24 (30.4%). Tumour grade was high grade in 62 patients (78.5%) and low grade in 17 (21.5%). Carcinoma in situ was identified in three patients (3.8%) at index resection and again in three patients (3.8%) at restaging.

**TABLE 1:** Baseline demographics and tumour characteristics.

Domain	Variable	Category or summary	Value
Demographics	Total patients		79
	Age (years)	Median (interquartile range)	64 (56 to 69)
	Sex	Male	69 (87.3%)
		Female	10 (12.7%)
Outcome	Repeat transurethral resection result	Positive	9 (11.4%)
		Negative	70 (88.6%)
Tumour pathology	Index stage	Stage Ta	10 (12.7%)
		Stage T1	69 (87.3%)
Tissue quality indicator	Detrusor muscle in the index specimen	Present	77 (97.5%)
		Absent	2 (2.5%)
Tumour burden	Tumour size category	<3 cm	46 (58.2%)
		≥3 cm	33 (41.8%)
	Tumour multiplicity	Single	55 (69.6%)
		Multiple	24 (30.4%)
Histopathology	Tumour grade	High grade	62 (78.5%)
		Low grade	17 (21.5%)
High‐risk pathology	Carcinoma in situ on index histopathology	Present	3 (3.8%)
		Absent	76 (96.2%)

A positive restaging histopathology (defined as residual urothelial neoplasia and/or pathological progression/upstaging, compared with the index specimen) was observed in nine of 79 patients (11.4%). Of these, eight patients (10.1%) had residual disease, and one patient (1.3%) had progression. The positive restaging group included patients with persistent high‐grade disease and patients in whom carcinoma in situ was identified on restaging. No patient demonstrated confirmed muscle‐invasive disease on restaging histopathology.

On exploratory univariable comparison, positive restaging histopathology was more frequent among patients with multiple tumours than among those with a single tumour: 6 of 24 (25.0%) versus 3 of 55 (5.5%); unadjusted odds ratio 5.78, 95% confidence interval 1.31–25.53; Fisher's exact *p* = 0.02. Positive restaging histopathology was also more frequent among patients with tumours ≥3 cm than among those with tumours <3 cm: seven of 33 (21.2%) versus 2 of 46 (4.3%); unadjusted odds ratio 5.92, 95% confidence interval 1.14–30.67; Fisher's exact *p* = 0.03. These comparisons were exploratory and should not be interpreted as definitive predictors.

Age and sex did not show meaningful differences between groups. When analysed as a continuous variable, age per 10‐year increase had an unadjusted odds ratio of 1.12 (95% confidence interval 0.56–2.22; *p* = 0.75). Age was additionally dichotomised at >70 years because age above 70 years is recognised in contemporary NMIBC risk‐stratification frameworks as an additional clinical risk factor. When analysed using this clinically relevant threshold, positive restaging histopathology was observed in two of 14 patients (14.3%) aged >70 years and in seven of 65 patients (10.8%) aged ≤70 years, with no meaningful difference between groups (unadjusted odds ratio 1.38, 95% confidence interval 0.26–7.37; *p* = 0.66). Positivity was noted in eight of 69 males (11.6%) and 1 of 10 females (10.0%) (unadjusted odds ratio 1.18, 95% confidence interval 0.13–10.58; *p* = 1).

CIS on index histopathology was uncommon, but positive restaging histopathology was observed in two of three patients with CIS compared with seven of 76 without CIS. The unadjusted odds ratio was 19.71, but the 95% confidence interval was wide (1.58–245.82; *p* = 0.03), reflecting the very small CIS subgroup. This finding should therefore be interpreted with particular caution. Exploratory univariable comparisons according to restaging histopathology are presented in Table 2.

**TABLE 2:** Exploratory univariable comparison of clinicopathological variables according to restaging histopathology.

Predictor	Comparison	Positive proportion	Effect size	*p* value
Age	Per 10‐year increase	Not applicable	Odds ratio 1.12 (95% CI 0.56 to 2.22)	0.75
Age	>70 years compared with ≤70 years	2 of 14 compared with 7 of 65	Odds ratio 1.38 (95% CI 0.26 to 7.37)	0.66
Sex	Males compared with females	8 of 69 compared with 1 of 10	Odds ratio 1.18 (95% CI 0.13 to 10.58)	1
Tumour size category	≥3 cm compared with <3 cm	7 of 33 compared with 2 of 46	Odds ratio 5.92 (95% CI 1.14 to 30.67)	0.03
Tumour multiplicity	Multiple compared with single	6 of 24 compared with 3 of 55	Odds ratio 5.78 (95% CI 1.31 to 25.53)	0.02
Carcinoma in situ on index histopathology	Present compared with absent	2 of 3 compared with 7 of 76	Odds ratio 19.71 (95% CI 1.58 to 245.82)	0.03

Follow‐up was calculated from the date of restaging TURBT to the last available institutional record. The median follow‐up duration was 20.1 months (IQR 10.8–34.1). During follow‐up, recurrent bladder tumour admission after re‐TURBT was documented in nine of 79 patients (11.4%). Recurrence occurred in one of nine patients (11.1%) with positive restaging histopathology and in eight of 70 patients (11.4%) with negative restaging histopathology. The only patient with positive restaging histopathology who later recurred developed recurrence 31.0 months after re‐TURBT. These outcomes were interpreted descriptively because surveillance schedules and intravesical treatment completion were not uniformly available for all patients.

## 4 DISCUSSION

In this single‐centre cohort from a tertiary referral unit, the rate of positive findings at restaging transurethral resection of bladder tumour was low, with positivity in 11.4% and progression uncommon. These results are clinically important because repeat resection remains strongly recommended for stage T1 non‐muscle‐invasive bladder cancer in major contemporary guidelines, primarily on the basis of historical evidence demonstrating substantial residual tumour and clinically meaningful upstaging after the index procedure.^1^, ^2^, ^3^ The European Association of Urology continues to advise repeat resection for stage T1 disease, for incomplete resections, and for tumours with absent detrusor muscle.^1^ Similarly, the American Urological Association and Society of Urologic Oncology guideline emphasises timely repeat resection for stage T1 disease, typically within six weeks.^2^ The National Comprehensive Cancer Network also endorses repeat resection in stage T1 disease and other high‐risk scenarios, reflecting the potential impact of residual disease on recurrence and subsequent management decisions.^3^

When placed against globally reported outcomes, the present yield is lower than many historical benchmarks but consistent with the direction of more contemporary series in settings where index resection quality is optimised. The early 2000s series reported substantial residual disease. In the 2003 long‐term observational study by Grimm and colleagues, residual tumour was detected in 33% overall, including 53% in stage T1, and tumour was most often located at the initial resection site, implying incomplete index surgery as a major contributor to early recurrence.^4^ This observation is consistent with the broader evidence that residual tumour following repeat resection is common and frequently located at the original site.^5^ In a later influential institutional series (patients diagnosed between 1990 and 2007; published 2009), Dalbagni and colleagues reported upstaging to muscle‐invasive disease in 20% of restaged patients and highlighted a key quality signal: detrusor muscle was present in only 47% of index specimens but 84% at restaging, underscoring how staging accuracy improves when deep muscle is consistently sampled.^6^

Moving into the 2010s, several cohorts began to show lower upstaging rates and more nuanced risk stratification. Bishr and colleagues reported that pathological stage at restaging stratified prognosis in high‐risk non‐muscle‐invasive bladder cancer, with patients staged as T0 at restaging having markedly better recurrence‐free and progression‐free outcomes than those with persistent disease.^7^ This reinforces the notion that restaging may serve as a risk‐stratification tool in high‐risk disease rather than merely as a completion procedure.^7^ The clearest evidence for temporal change comes from contemporary synthesis. In the updated systematic review and meta‐analysis in the contemporary era (published 2024), Yanagisawa and colleagues reported that among stage T1 patients treated with conventional transurethral resection in the 2010s, pooled residual tumour and upstaging rates at repeat resection were 31.4% and 2.8%, respectively, and these rates were significantly lower than those reported in the 1990–2000s.^8^ Their analysis explicitly frames improving resection quality and modern procedural developments as drivers that may permit omission of repeat resection in selected patients, while retaining restaging for those at highest risk.^8^ Although pooled contemporary estimates remain higher than our observed positivity, the temporal direction is concordant: Globally, clinically meaningful positive outcomes, particularly upstaging, appear to be decreasing over time as surgical technique and quality metrics improve.^7^, ^8^

This trend is also reflected in the heterogeneity of earlier‐era cohorts. The 2018 systematic review by Cumberbatch and colleagues reported detrusor muscle presence in index specimens varying widely, residual tumour in 20%–71% for stage T1 disease, and upstaging from stage T1 to at least stage T2 reported up to 32% in some series, reinforcing that repeat resection yield is heavily contingent on baseline resection adequacy and pathological reporting.^5^ In parallel, earlier single‐centre experiences also argued that repeat resection can detect residual disease even in experienced hands, supporting the original rationale for routine restaging in stage T1 disease.^4^, ^5^, ^6^, ^7^ In contrast, contemporary cohorts increasingly emphasise the quality of index resection as the key determinant of whether routine repeat resection adds incremental staging value.^8^, ^9^

Against this global backdrop, the low positivity and absence of muscle‐invasive upstaging in our cohort likely reflect a high‐quality index resection environment, particularly the near‐universal inclusion of detrusor muscle in index specimens. The detrusor muscle in the initial transurethral resection specimen has been repeatedly proposed as a pragmatic surrogate marker of resection quality and depth. Mariappan and colleagues demonstrated that the presence of detrusor muscle in apparently complete resections was associated with improved early outcomes and reflected operator experience.^9^ More recent work has supported the prognostic relevance of detrusor muscle absence and has explored predictors of missing muscle and its clinical impact.^10^ When the detrusor muscle is consistently captured and the tumour base is deliberately resected, the principal benefit of routine restaging may diminish for low‐enrichment groups, particularly when tumour burden is limited and pathological uncertainty is low.^8^, ^9^, ^10^

A central objective of this study was to identify clinical determinants that can guide selectivity. Earlier single‐centre experiences also argued that repeat resection can detect residual disease even in experienced hands, supporting the original rationale for routine restaging in stage T1 disease.^11^, ^12^ In our analysis, multiplicity at index resection emerged as the most robust determinant of positivity, with a markedly higher positivity rate in multifocal tumours. Multifocality is a recognised marker of field change and higher recurrence risk and is incorporated into established risk stratification models such as the European Organisation for Research and Treatment of Cancer and Club Urológico Español de Tratamiento Oncológico scoring systems.^13^, ^14^ Tumour size similarly influences recurrence risk and is embedded in these tools, reflecting both biological aggressiveness and the technical challenges of complete resection.^13^, ^14^ In our cohort, tumours at least three centimetres and multiple tumours were associated with significantly higher odds of positive restaging, which is consistent with these global prognostic constructs.^13^, ^14^ Carcinoma in situ, although uncommon in our cohort, also showed a strong clinical association with positivity; this is biologically plausible and consistent with guidelines that treat carcinoma in situ as a defining high‐risk feature influencing intravesical therapy and surveillance intensity.^1^, ^2^, ^3^, ^13^, ^14^

Technological and procedural developments may also be contributing to the global downward trend in positive restaging outcomes. En bloc resection has been associated with improved specimen integrity, greater detrusor muscle identification, and reduced residual tumour in several analyses; randomised trial meta‐analyses suggest favourable perioperative outcomes and potential oncological advantages.^15^ Optical enhancement, particularly photodynamic diagnosis, improves tumour detection and has been associated with better recurrence‐related outcomes in meta‐analyses, plausibly by enabling more complete initial resection and the identification of occult lesions.^16^ These developments support the conceptual shift towards defining high‐quality index resection and using it to develop selective restaging pathways rather than applying repeat resection uniformly.^8^, ^15^, ^16^

Despite supportive signals for selectivity, the rationale for restaging remains strong in enriched‐risk groups because residual disease is not eliminated in modern practice. Contemporary pooled analyses still report nontrivial residual tumour and upstaging rates in stage T1 cohorts, and the oncological advantage of routine repeat resection, as shown in older prospective evidence, cannot be ignored.^4^, ^5^, ^6^, ^7^ Furthermore, some contemporary studies continue to demonstrate meaningful yields in specific settings, including high‐grade Ta disease, where repeat resection remains debated, and in selected series where restaging changed management.^17^, ^18^ Importantly, emerging contemporary data also suggest that a second resection may be safely omitted in carefully selected patients with primary high‐grade Ta non‐muscle‐invasive bladder cancer after complete index resection with adequate deep muscle sampling.^19^, ^20^ In a 2025 study by Abe and colleagues evaluating primary high‐grade Ta disease, residual tumour and upstaging at second resection were low, and oncological outcomes were comparable between patients who underwent a second resection and those who did not, supporting a selective rather than routine approach in appropriately chosen cases.^21^ Therefore, our results should be interpreted as evidence that yield may be concentrated in enriched‐risk subgroups, not as a rationale for indiscriminate restaging omission.

The limitations of this study include its retrospective design, single‐centre setting, and small number of positive outcomes. Comparisons across decades must also be interpreted with caution because case mix, staging definitions, use of intravesical therapy, and surveillance schedules vary across eras and institutions. Nonetheless, by situating our low contemporary positivity rate within year‐wise global evidence, from higher residual and upstaging rates in older cohorts to lower pooled upstaging and lower residual disease in the 2010s, we provide centre‐specific support for the question posed by this manuscript: whether modern practice can move from routine to selective restaging in appropriately defined low‐enrichment groups, while preserving restaging for those at highest risk of residual disease or progression.

## 5 CONCLUSIONS

In this contemporary re‐TURBT cohort, residual/recurrent tumour detection and pathological progression were uncommon, with no muscle‐invasive upstaging. Positive restaging findings were enriched in patients with larger tumours, multifocal disease, and CIS on index histopathology, whereas age and sex did not differ meaningfully between groups. These findings are hypothesis‐generating and support prospective evaluation of selective, risk‐adapted restaging strategies while maintaining guideline‐concordant vigilance for high‐risk NMIBC.

## AUTHOR CONTRIBUTIONS

Mithilesh Yadav contributed to idea and study conception, data collection, data analysis, manuscript drafting, and revision. Swarnendu Mandal contributed to study conception, methodology, supervision, interpretation of data, and critical revision of the manuscript. Sambit Tripathy contributed to methodology, clinical supervision, interpretation of results, and critical revision. Pranjal Chowdhury contributed to data collection, data verification, literature review, and manuscript revision. Vivek Rathod contributed to data collection, data verification, and manuscript revision. Manoj Kumar Das contributed to clinical supervision, interpretation of data, and critical revision of the manuscript. Kalandi Barik contributed to clinical supervision, interpretation of data, and critical revision of the manuscript. Prasant Nayak contributed to overall supervision, study oversight, interpretation of findings, and final critical revision of the manuscript. All authors reviewed and approved the final manuscript and agree to be accountable for all aspects of the work.

## CONFLICT OF INTEREST STATEMENT

The authors declare no conflicts of interest.

## Data Availability

The data that support the findings of this study are available from the corresponding author upon reasonable request.
